# Assessing radiological hazards in building materials: a case study with a focus on the ceramic industry in Emilia-Romagna, Italy

**DOI:** 10.1007/s11356-025-36178-w

**Published:** 2025-03-05

**Authors:** Laura Laghi, Alessandro Zappi, Domiziano Mostacci, Laura Tositti

**Affiliations:** 1 Department of Industrial Engineering, University of Bologna, Via Dei Colli 16, 40136 Bologna, Italy; 2 Department of Chemistry, University of Bologna, Via Gobetti 85, 40129 Bologna, Italy

**Keywords:** NORM, Tiles, Bricks, Building materials, Dosimetric index, HPGe gamma-ray spectrometry

## Abstract

The building materials industry encounters naturally occurring radioactive materials problems and elicits growing attention in radiation protection regulations. However, the availability of useful, comprehensive data on radiological hazard in building materials is unfortunately scant: data are few and far between. In the Italian region of Emilia-Romagna, there is a flourishing ceramic industry, with a vast production of building materials, particularly tiles and bricks. Our laboratory of Environmental Chemistry and Radioactivity has collaborated with this industry since the year 2000, collecting over time a vast number of samples and processing them through high-resolution gamma spectrometry, to obtain a large dataset of radionuclide contents. This paper presents a radiation protection study based on said dataset, aimed at assessing the radiological risk associated with these materials: in particular, different indexes, internationally accepted, and dose rates are calculated in compliance with well-established EU algorithms. Statistical treatment of data is also presented.

## Introduction

Naturally occurring radioactive materials (NORM) in building materials are associated with the ubiquitous occurrence of radioactive families of ^238^U, ^235^U, ^232^Th, and primordial ^40^K in all lithogenic materials. NORM contributes a large fraction of the overall natural radiation exposure (Nazaroff and Nero 1988; Eisenbud and Gesell 1997; UNSCEAR 2008; Schön 2015a). This geogenic radioactivity is encountered in all building materials, from primary ones, historically used since antiquity, to secondary ones, such as bricks, tiles, cement, concrete, and all those derived from geological mixtures of soils, clay, sand, and gravel (Capaccioni et al. 2012; Eštoková and Palaščáková 2013; Schroeyers 2017; Benà et al. 2022). In recent decades, a new generation of building materials with potential radiological concern has emerged, based on recycled industrial solid wastes, e.g., coal fly ash, metal slags, and ceramic and brick waste from conventional building production chains (Coletti et al. 2020; Tositti et al. 2023).

NORM radiation protection issues emerged soon after the Chernobyl accident. This event contributed to raising awareness on all sources of radiation exposure beyond those associated with nuclear sources sensu stricto, progressively extending to the natural ones, and eventually calling for suitable regulation. In Europe, early attempts to regulate radioactivity in building materials appeared in 1999, when the European Commission published “Radiation Protection 112 (from now on RP112) – Radiological Protection Principles Concerning the Natural Radioactivity of Building Materials”—a guideline containing criteria and application rules aiming to establish a standardized dose assessment in this field (European Commission: Directorate-General for Environment 2000). To estimate exposure from building materials, RP112 has introduced the I-index, calculated from ^226^Ra, ^232^Th, and ^40^K activity concentrations measured experimentally using high-resolution gamma-ray spectrometry.

International awareness of potential radiation hazard from building materials has grown steadily. Although the NORM problem was recognized at the EU (European Union) level with Council Directive 96/29/EURATOM, only recently the EU concerned itself specifically with building materials within Council Directive 2013/59/Euratom (European Commission 2014). This regulation has extended the need for radiation protection regulations to a wider field of NORM hazard and management (European Commission 2014). The protection against natural radioactivity has become crucial across several environmental disciplines as well as productive fields, and many decades of interdisciplinary research in this area have led to the flourishing of international conferences in the field, e.g., the recent editions of IAEA’s *NORM 2020* (IAEA 2022) and *RadoNorm* (Kulka et al. 2022). The focus of the latter was on the wide occurrence of NORM across various industrial sectors and on new dosimetric metrology and standards for exposure assessment (Michalik et al. 2023).

Natural radioactivity in building materials yields one of the fundamental contributions to the average annual radiation dose to the population (ICRP 2007; UNSCEAR 2008), both in terms of external irradiation from the material, and of internal exposure due to radon exhalation and indoor buildup (Nazaroff and Nero 1988; European Commission: Directorate-General for Environment 2000; Schroeyers 2017). The quantitative assessment of the radiation gamma dose and radon progeny concerned is usually based on the determination of radium (^226^Ra), from the ^238^U series, thorium (^232^Th), and potassium (^40^K) by high-resolution gamma-ray spectrometry (IAEA 2010). Radon (^222^Rn), which is the immediate decay product of ^226^Ra, and thoron (^220^Rn from the ^232^Th family), along with their daughters, are historically recognized among the main causes of lung cancer in both smokers and non-smokers through their overall alpha emissions, following inhalation (Cinelli et al. 2015b, 2015a; Schroeyers 2017).

Concerning Italian regulation, it is important to mention that, despite the publication of D.L. 101 on July 31, 2020 (Parlamento Italiano 2020), the transportation of the Directive 2013/59 Euratom of December 5, 2013, its implementation is still missing. The Italian ceramic tile industry, which represents a remarkable asset in the national building materials scenario, with a high international ranking, has become aware of the raising radiological concern since the early 2000s. This stemmed from the need to comply with regulations in the global market and, i.e., with laws and screening values set by radiation protection standards and legislations in importing countries (Maringer et al. 2005).

Following current international guidelines on NORM in building materials, many countries have introduced activity-based indexes used as screening tools to ensure that indoor exposure does not exceed reference levels (Maringer et al. 2005; Sas et al. 2019). The attention towards indoor exposure is based on the average time people spend indoors—around 90% for average European citizens according to WHO (WHO 2014)—and on the indoor buildup of ^222^Rn brings in about the known exposure concerns (IAEA 2015). Outdoor exposure by building material NORM may be neglected due to the relatively shorter exposure, and to assimilation with the geogenic radioactive background. The latter is known to be highly variable and stochastically fluctuating in terms of environmental exposure contribution (see, for example, the recent EU digital maps to get an overview of the spatial distribution of NORM, useful also for lithogenic radioactivity appreciation (https://remap.jrc.ec.europa.eu/, visited July 2, 2024; (Tositti and Sogni 2007)).

One major challenge in collecting data on NORM and NORM-containing construction materials, besides data quality, is that literature is scattered in a number of small-scale/size data sources (Righi and Bruzzi 2006; Ortiz et al. 2008; Sas et al. 2017; Trevisi et al. 2018; Schroeyers et al. 2018). Such condition reflects the vast compositional variability of crustal materials even within the same lithogenic classes (Tositti et al. 2023), compounded with the globalized trade of raw materials and the influence of recycled waste. There are only a few comprehensive datasets available, all of which were organized manually, in large part reflecting regional geologies and national productive systems. Schroeyers et al. (2018) created a database by collecting data on raw materials, while Trevisi et al. (2018) gathered extensive data on activity concentration measurements of natural radionuclides (^226^Ra, ^232^Th, and ^40^ K) in building materials used in 26 European countries within the framework of COST Action TU1301—NORM for Building materials (Schroeyers 2017). Though this represents an improvement, database extensions and updates are always highly desirable, for better comparability and more accurate dose assessments.

In 2024, the association of Italian ceramic producers, Confindustria Ceramica, reported that in the previous year, ceramic tiles, sanitary ware, tableware, refractory materials, and bricks generated a total revenue of € 7.5 billion (Confindustria Ceramica 2024), 75% of which being export sales. This confirms the establishment of a flourishing sector, composed of 252 industrial companies, with 80% of the production sites located in the Emilia-Romagna region. In contrast to the only recent assimilation of NORM radiation protection regulations in the EU with their introduction into national legislation, ceramic tile industry in Italy had an early awareness of this issue and has long adopted radiation protection standards in the field of building materials because of export constraints. Regulations were already active in single countries as early as around 2000 (Mossini et al. 2015), forcing the early conformity of the production chain to appropriate NORM activity concentrations. A large part (albeit not all) of the long-term measurement activity reported in this article concerns the tile radioactivity assessment, as well as the characterization of raw and intermediate materials for suitable mixture design prior to production, obtained in this specific framework.

The goal of the present paper is to provide an overview of the radiometric content of a wide selection of tiles and other building products, as well as of raw and intermediate materials, and to evaluate the ensuing radiological impact. The assessment of natural radiation dose has been provided by using some of the most frequently used radiological parameters—activity concentration index, alpha index, radium equivalent activity, annual external dose rate, and internal and external hazard indices—for building material screening that can be found in literature (Trevisi et al. 2013; Khatun et al. 2018). Further aim of this work is to contribute to expanding the European dataset of radiometric parameters of building and construction materials and to evaluate the potential radiological hazards associated with the NORM presence in such materials, covering both raw materials and final products.

## Materials and methods

### Dataset

The Laboratory of Environmental Chemistry and Radioactivity (ACERLAB-University of Bologna) possesses long-term experience in radioactivity analysis in a range of environmental and technological samples. These include building materials and raw materials. A systematic assessment of compliance with non-EU radiation protection regulation has been carried out, for many companies of the Emilia Romagna ceramic district, since 2010. The total number of samples to date is around 500, more than 70% of which are tiles of all types of manufacturers: from minor artisan companies up to large hi-tech industrial realities. Data were classified into seven main categories: tiles (365 samples), bricks (50 samples), atomized materials^1^(30samples), sands (20 samples), zircon sands (15 samples), clays (12 samples), and granites (8 samples). NORM analyses were carried out with the aim of determining the final radioactivity concentration range for each composite building material, but also to single out potentially critical contributions emerging from each of the raw materials before mixing. “Granite” are primary rock materials that undergo reduced or no processing between mining and use.

### HPGe spectrometry and radionuclide composition

High-resolution gamma-ray spectrometry by germanium detectors (HPGe) is a golden standard for the quali-quantitative analysis of radioactivity and, consequently, activity concentration levels of NORM in tiles, bricks, and raw materials. Samples were analyzed with a p-type HPGe coaxial detector (PROFILE, AMETEK Inc., Oak Ridge, TN, USA) with an extended energy range (20–2000 keV). This detector has a relative efficiency of 38%, and resolution (FWHM) at 1332.5 keV of 1.8 keV. The calibration of the system was made with a liquid standard source (Eckert & Ziegler Multinuclide standard solution 7501) in a jar geometry (diameter = 56 mm; thickness = 10 mm). After 1 day of acquisition time, sample spectra were processed with the software Gamma Vision-32, version 6.08 (AMETEK Inc., Oak Ridge, TN, USA). The radionuclides are identified and quantified using a NORM-based isotopic library including the emission of the most relevant gamma emitters of the three natural radioactive families (^238^U, ^235^U, and ^232^Th, plus primordial ^40^K, chosen based on the highest gamma decay probability (yield)). ^226^Ra was determined at 186 keV correcting the peak area by the ^235^U interference, under the hypotheses of secular equilibrium between ^226^Ra-^238^U and natural ^235^U/^238^U isotopic ratio, according to the method proposed by Gilmore (Gilmore 2008). This prevents the waste of time required for reaching secular equilibrium among ^214^Pb and ^214^Bi and the use of gas tight counting containers to prevent radon leaks.

This method calculates U concentration from the ^214^Pb and ^214^Bi isotopes, not from the convenient ^234^Th γ emission at 63.3 keV, as a suitable self-absorption correction is not available at our lab yet. On the other side, the 1001 keV line of ^234 mPa^ has a very weak yield (emission probability), not compatible with the small size of the sample geometry we have traditionally adopted at our lab and that thanks to lower efficiency of HPGe in this energy range lead to poor detectability (Lenka et al. 2009) not to mention the huge variability the yield % shows the across the γ lines libraries, thus preventing the assessment of the ^238^U/^226^Ra equilibrium conditions. Additionally, ^235^U lines are very uncertain for the well-known interferences of its emissions, limiting the reliability of ^238^U extrapolation on the basis of natural U isotopic ratio.

Due to what previously described, we treated ^226^Ra equilibrium pragmatically at our lab. We therefore assumed that ^238^U-^226^Ra reasonably was in secular equilibrium as the ^234^Th levels, though not usable due to the lack of self-absorption correction (and therefore not reported) shows values intuitively compatible with the equilibrium in the upper part of the decay chain. Overall, the corrected ^226^Ra activity concentration obtained in our lab by Gilmor’s algorithm yields values systematically ≥ ^214^Bi, ^214^Pb, though reasonably close on account of the overall uncertainty, indicating the tendency to reach the corresponding experimental ^226^Ra level. Moreover, the type of samples treated in this work is basically of mineral origin (mixtures of clays, kaolin, and ZrSiO_4_ in the case of tiles) with a reduced risk of differential leaching of some members of the U and Th families, due to differential aqueous solubility and consequent departure from secular equilibrium between parent nuclides and the corresponding radium isotopes, a risk instead expected for environmental samples and waste-derived materials. The lack of radioactive equilibrium between the parent ^226^Ra and the daughters is expected due to the type of polystyrene container routinely used for counting, which is not gas-tight, implying the loss of radon and the departure from equilibrium. The use of Gilmore’s corrected ^226^Ra, though refinable, at least allows a dosimetric assessment closer to reality (and therefore more precautionary) than the most widely used approach based on the use two 214-mass γ-emitters. As a rule, gas-tight containers are costly and not easily available, moreover in the lab waste management amount and type of samples may be fairly hard to dispose of, thus suggesting aware but pragmatic practice.

The minimum detectable activity (CRMDA) was calculated according to the Traditional ORTEC method available in Gamma Vision-32 (AMETEK Inc., Oak Ridge, TN, USA) using the following equation:

$${CR}_{MDA}= \frac{\frac{100}{SENS}\left(\sqrt{2\cdot {B}_{1}+\frac{2500}{{SENS}^{2}}}+\frac{50}{SENS}\right)}{LT}$$

where SENS is the peak cutoff value (%) on the analysis tab, B_1_ is the peak background, and LT is the live time (s).

Measurement quality control has been carried out systematically using certified reference materials such as UTS3 and DH1a by CANMET and IAEA-412.

The activity concentrations of ^226^Ra (A_Ra_), ^232^Th (A_Th_), and ^40^ K (A_K_), expressed in Bq/kg are calculated from gamma spectra. Then, range and mean values with respective standard deviation are calculated for each of the seven material classes.

### Multivariate analysis

In this paper, activity concentration data and the indexes calculated were subjected first to basic statistical processing, then to multivariate analysis, i.e., principal component analysis (PCA) with the aim of revealing correlation patterns among a sufficiently large dataset, based on data reduction techniques.

PCA (Bro and Smilde 2014) is a well-known chemometric technique based on the computation of a linear combination of the original variables (NORM activities in this case). This mathematical procedure rotates the spaces spanned by the original variables to obtain a new space whose first unit vectors (the principal components, PCs) carry most of the original information (explained variance, EV%). Using this method, 2D or 3D plots of samples (scores plot) and variables (loadings plot) can fully describe the problem in terms of similarity or dissimilarity of both samples and variables, and which are the variables that mostly influence the samples distribution (Zappi et al. 2023). In this work, PCA was performed on the radionuclide activities of the comprehensive dataset with the statistical software R (R Core Team 2021), using a tailored in-house script.

### Radiological parameters

Based on the activity concentrations of ^226^Ra (A_Ra_), ^232^Th (A_Th_), and ^40^K (A_K_), expressed in Bq/kg and calculated from gamma spectra, several activity indexes have been calculated for all samples. Table 1 reports the algebraic expressions used for the calculation of each parameter, followed by a short description.

**Table 1:** Definition of the radiological parameters herein used

Radiological parameter		Definition	Reference
*Activity concentration index*	**I**_**γ**_	${I}_{\gamma }=\frac{{A}_{Ra}}{300 Bq/kg}+\frac{{A}_{Th}}{200 Bq/kg}+\frac{{A}_{K}}{3000 Bq/kg}$	European Commission: Directorate-General for Environment (2000)
*Alpha index*	**I**_**α**_	${I}_{\alpha }=\frac{{A}_{Ra}}{200 Bq/kg}$	Righi and Bruzzi (2006)
*Radium equivalent activity*	**Ra**_**eq**_** [Bq/kg]**	${Ra}_{eq}={A}_{Ra}+1.43 {A}_{Th}+0.077{A}_{K}$	Beretka and Mathew (1985)
*Absorbed dose rate*	**D**_**R**_** [nGy/h]**	${D}_{R, mat}=0.92 {A}_{Ra}+1.1 {A}_{Th}+0.08 {A}_{K}$	European Commission: Directorate-General for Environment (2000)
		$${D}_{R, tile}=0.12 {A}_{Ra}+0.14 {A}_{Th}+0.0096 {A}_{K}$$	
*Annual effective dose rate*	**AEDR [mSv/y]**	$AEDR ={D}_{R}\times 8760 \frac{h}{y}\times 0.7 \frac{Sv}{Gy}\times {10}^{-6}\times 0.8$	European Commission: Directorate-General for Environment (2000)
*Internal hazard indexes*	**H**_**in**_	${H}_{in}=\frac{{A}_{Ra}}{180 Bq/kg}+\frac{{A}_{Th}}{259 Bq/kg}+\frac{{A}_{K}}{4810 Bq/kg}$	Beretka and Mathew (1985)
*External hazard indexes*	**H**_**ex**_	${H}_{ex}=\frac{{A}_{Ra}}{370 Bq/kg}+\frac{{A}_{Th}}{259 Bq/kg}+\frac{{A}_{K}}{4810 Bq/kg}$	Beretka and Mathew (1985)

Over the past 10 years, the most common screening tool (Nuccetelli et al. 2012; Ignjatović et al. 2017) used to identify building materials that are of concern from the radiological protection point of view is the ${I}_{\gamma }$ index. It was introduced in RP112 (European Commission: Directorate-General for Environment 2000), where the idea was to give a practical way to indicate whether the annual dose due to the excess of external gamma radiation in a building could exceed 1.0 mSv. RP112 recommended two threshold values, ${I}_{\gamma }$ ≤ 1 and ${I}_{\gamma }$ ≤ 0.5, which correspond to an absorbed gamma dose rate less or equal to 1 mSv/y and 0.3 mSv/y, respectively.

The excess alpha radiation due to radon inhalation originating from building materials is assessed through the alpha index, which indeed highlights the risk that can be brought by radon contribution alone.

The radium equivalent activity is used to represent the activity levels of ^226^Ra, ^232^Th, and ^40^K using a single quantity that considers the associated hazards. In this common radiological index, it has been assumed that 10 Bq/kg of ^226^Ra, 7 Bq/kg of ^232^Th, and 130 Bq/kg of ^40^K will induce the same dose of gamma radiation. The limit value of Ra_eq_ in building materials is 370 Bq/kg. This index was widely used (Ibrahim 1999) before defining the activity concentration index ${I}_{\gamma }$ in 1999.

Following the guidelines provided by UNSCEAR (UNSCEAR 2000), absorbed dose rates and annual effective dose rates due to gamma radiation have been calculated. The specific dose rate values are provided in RP112 (European Commission: Directorate-General for Environment 2000), both for structures and for superficial materials. The annual external effective dose rate can be estimated from D_R_, using the conversion factor from absorbed dose in air to effective dose (0.7 Sv/Gy) and the indoor occupancy factor (0.8) suggested in (European Commission: Directorate-General for Environment 2000).

In addition, internal and external hazard indexes have been introduced to compare them with the alpha and the activity concentration indices, respectively. To safely use a building material, the excess internal radiation exposure caused by inhalation of ^222^Rn and its short-lived daughters originating from NORM should be less than unity (Krieger 1981). As well as for the internal doses, also the limit value beyond which the external exposition could become a potential risk to the public is the unity value.

## Results and discussions

### Statistical analysis of radionuclide composition data

Table 2 summarizes the main characteristics of the 500 samples so far analyzed at ACERLAB. The samples reported are split into seven subsets, i.e., tiles, bricks, and several raw materials such as clays, sands, zircon sands, and granites as well as intermediates of tile production chains such as the “atomized materials.” Table 2 reports their range of activity concentrations for the three NORMs used for the index computation, namely ^226^Ra, ^232^Th, and ^40^K. The complete dataset, including all the determined radionuclides, will be made available subsequently as an independent article in a data-devoted journal.

**Table 2:** mean values and ranges of activity concentrations of natural radionuclide

Material	N	^226^Ra [Bq/kg]		^232^Th [Bq/kg]		^40^ K [Bq/kg]	
		Range	Mean ± SD	Range	Mean ± SD	Range	Mean ± SD
Brick	50	10–190	68 ± 32	31–89	54 ± 15	519–1263	725 ± 156
Tiles	365	25–257	97 ± 48	18–98	53 ± 13	50–1528	622 ± 211
Atomized	30	28–231	67 ± 43	19–78	50 ± 10	330–1394	629 ± 196
Sand	20	10–593	187 ± 233	6–130	60 ± 38	143–1240	596 ± 334
Zircon Sand	15	1675–6439	3133 ± 1485	353–782	546 ± 172	76–239	135 ± 53
Clay	12	36–91	63 ± 17	44–98	70 ± 17	232–925	556 ± 232
Granite	8	61–125	79 ± 21	50–89	72 ± 12	1146–2126	1401 ± 305

Bricks are mainly constituted by raw clay and sand mixtures; tiles are technologically more advanced, albeit the largest mass contribution comes from the same raw materials. Differences may come from mineral additives (e.g., granites, ZrSiO_4_, and pigments) and the types of industrial processes applied, e.g., the ones leading to the production of atomized materials, an intermediate mixture in tile production. The resulting radioactivity in all cases is the weighted mean of the radioactivity of the single components. The data collected by HPGe spectrometry reveals that bricks, clays, and sand show on average lower activity concentration values, rarely reaching the radiation protection threshold. This agrees with the typical radioactivity concentration range of raw materials because of the rock formation process (UNSCEAR 2008; Schön 2015b). The results obtained are comparable with values in literature (Trevisi et al. 2018; Imani et al. 2021).

**Fig. 1:**
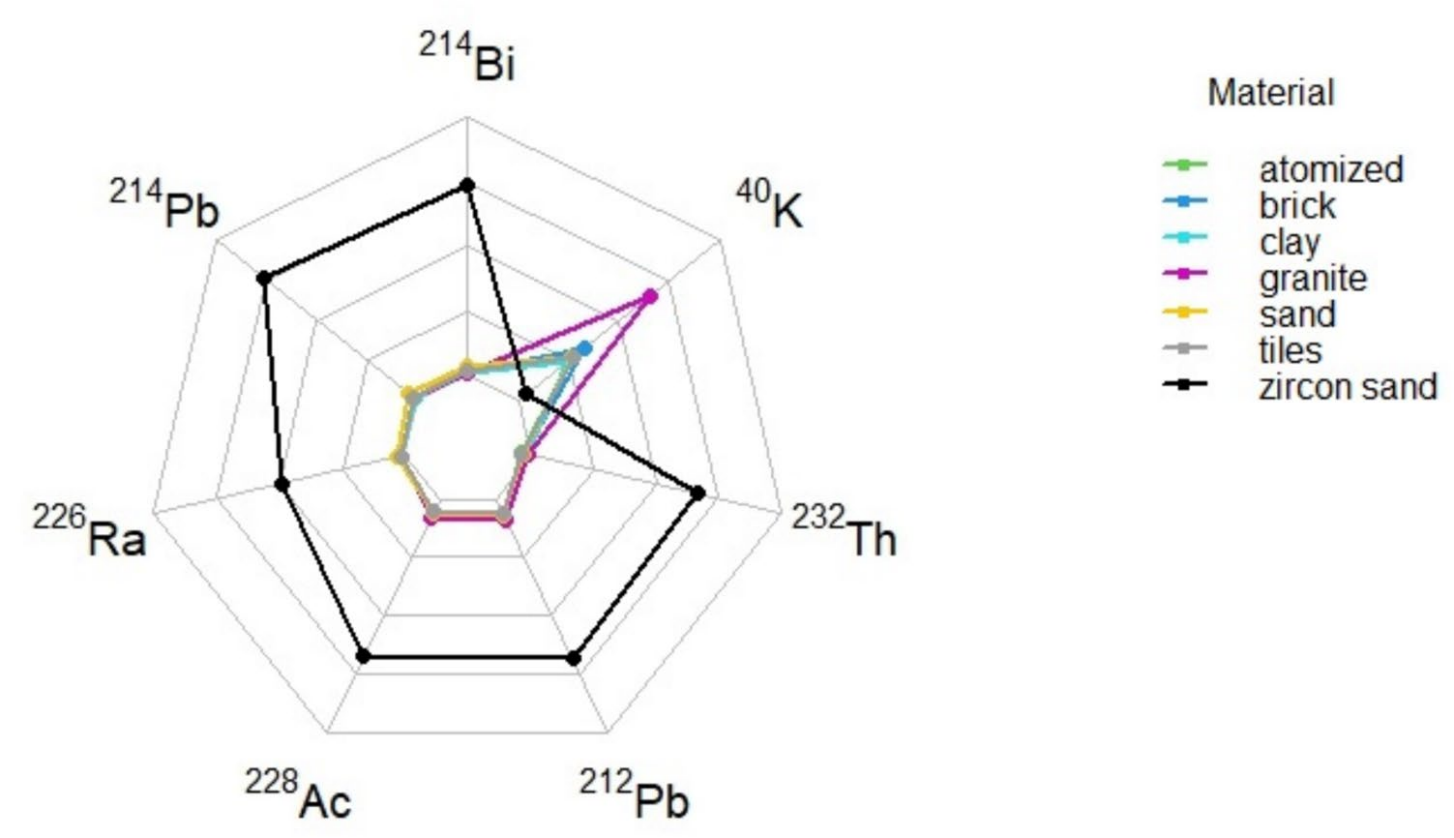
Radar chart of radionuclides composition for each material

Zircon sands are characterized by radioactivity concentrations significantly higher than all the other materials examined. As shown in Fig. 1, the mean activity concentration of ^226^Ra in zircon sand samples is several times higher than thorium and potassium contribution (the latter fairly depleted as compared to all the other lithogenic materials) and up to two orders of magnitude higher when compared to all the other materials (Damonte et al. 2017).

In terms of their mass concentrations, zircon sand samples have a mean value of 3133 ± 1485 Bq/kg of uranium compared to significantly lower ranges for all the other samples, while thorium concentration in zircon sands is roughly ten times that of all the other materials. The use of zircon silicates in ceramic industry has long represented a significant radiation protection issue leading to consolidated awareness of their radiological hazard on account of the wide use of this strategic material in a variety of industrial processes. Zircon sands have remarkably high NORM levels with a wide range of variability depending on source location and zircon formation process: the enrichment in uranium and thorium in this mineral is due to the allocation of uranium and thorium atoms in ZrSiO_4_ crystal lattice, due to similar ion size, thus promoting co-crystallization (Finch and Hanchar 2003). In the ceramic tile industry, zircon sand is used for glaze and ceramic mixtures. Though used in minor mass fractions, its high NORM content may result crucial in the final radioactivity level, and, if not accurately assessed and dosed in all the components, can easily lead to products above the safety threshold advised in radiation protection regulations.

Moreover, granites may constitute a problem since this family of metamorphic lithotypes tends to show NORM levels higher than average within rocks. Their use must be assessed carefully whether used as structural or surface stone material or when milled and mixed in composite materials.

Radiation hazard from building materials is related both to external and internal exposure with a γ irradiation term explicitly accounted for by the current I index, provided in the Euratom 2013 Directive and widely treated in this work, while the second contribution is related to radon emission potential from the NORMs contained in the building materials. While both the dose contributions refer to indoor conditions, the potential for radon emanation and buildup under this exposure scenario is highly dependent on (1) structural conditions, i.e., the discontinuity between soil and the building, (2) the intrinsic NORM content in the building material as determined by γ-ray spectrometry, and (3) the porosity of the building materials. It is to note, however, that, in case of an indoor ambience, a surface treatment of the walls/materials is usually applied, ranging from plaster and paint, typical of bricks, up to glaze applied directly on tiles during their production. In the former case, substrate porosity might be improved in respect to bricks by the covering layer, thus reducing somehow radon emanation power; the glaze over the tiles, instead, substantially blocks radon emanation power more efficiently owing to the glassy/vitreous nature of the glaze itself. Nevertheless, tile glaze has historically represented one of the main radiation hazards from tile production, due to the significant NORM enrichment in ZrSiO_4_ abundantly employed in this surface tile component, especially in the recent past (Damonte et al. 2017). However, emanation studies carried out in the past showed that glaze was able to efficiently inhibit radon degassing in Italian tiles, bringing emanation below the detection limit, thus ensuring their radon-safety compliance (Righi and Bruzzi 2006; Barescut et al. 2009b). As a final further remark, it is to note that the majority of the tiles herein reported are made in porcelain grés, whose production technology, aiming at reducing tile thickness/weight and porosity in order to enhance mechanical and staining resistance, is sought to eliminate radon exhalation not only from the surface, but from the whole tile body (Sánchez et al. 2010).

**Fig. 2:**
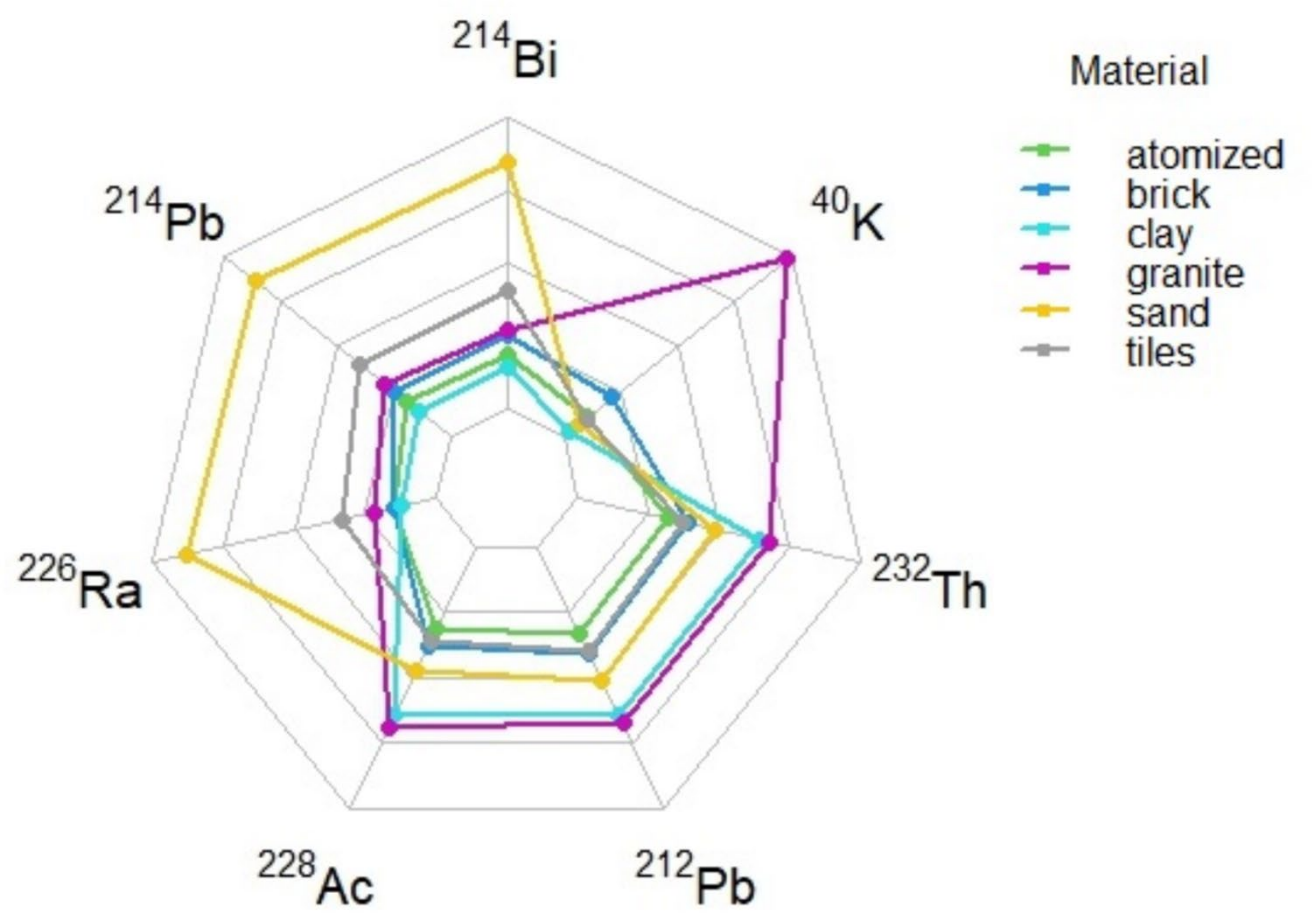
Radar chart of radionuclides for each material except zircon sand

Figure 2 visualizes the radionuclides average composition of each group of the dataset, besides zircon sand. As already mentioned in the previous paragraph, we can notice that ^40^K component of granites dominates with respect to the other materials, while sand has a higher contribution from ^238^U family. Bricks and tiles present a more balanced contribution from the different radionuclides, while ^232^Th family is more evident in clay samples.

### PCA of radionuclide composition data

**Fig. 3:**
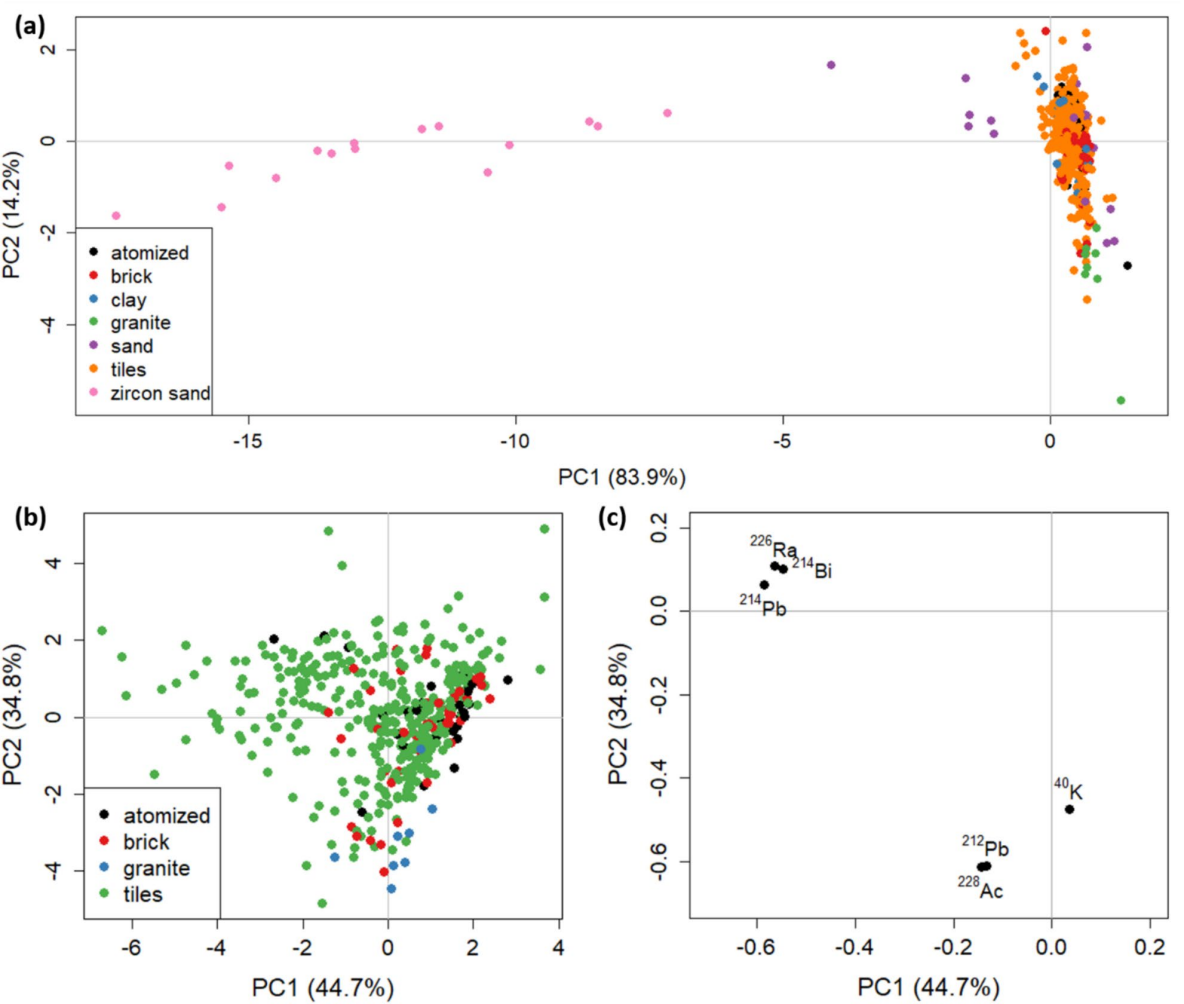
PCA calculated with the activity concentration of radionuclides: **a** scores for the entire dataset; **b** and **c** scores and loadings for the building materials dataset (without clay, sand, and zircon sand samples)

A first PCA was carried out on the dataset using all the radionuclides activities as variables (Fig. 3). The first two principal components, PC1 and PC2, reported in Fig. 3a substantially support the whole information contained in the dataset, with 98.1% of the total explained variance. Brick, tiles, and the raw + atomized materials are highly overlapped at the top right side of the plot. This is due to zircon sands, at highly negative PC1 values, among the generic raw materials with higher activity concentrations in most NORMs, as partially reported in Table 2. This brings the PCA model to sharpen their discrimination compared to the other materials, to the detriment of all other information. Therefore, in Fig. 3b (scores) and c (loadings), the analysis was performed by removing sand and zircon sand samples, focusing on the other (most numerous) groups of the dataset. In this case, the total explained variance carried by PC1 and PC2 is 77.8% and the scores plot (Fig. 3b) shows again a considerable overlap among samples, confirming that their composition is substantially similar. Figure 3c highlights the NORM variables, whose closeness in the graph indicates correlation between radionuclides. It is interesting to note correlations between NORMs belonging to the same family. ^226^Ra, ^214^Bi, and ^214^Pb, on the upper left corner, indeed, are part of the ^238^U family, while ^228^Ac and ^212^Pb derive from ^232^Th one. On the other hand, the primordial radionuclide ^40^K shows a lower correlation with the other variables.

The overlapping points between bricks and tiles implies that there is no significant difference between the two types of finished composite building materials in terms of NORM composition wherein clay and sand represent the bulk components for both classes. Interestingly, while bricks and tiles show overlapping patterns, their distributions appear highly scattered due to the high mineralogical variability. Recalling how the terms clay and sand refer to the grain-size of these raw materials, their mineralogy is highly variable as a function of the primary rocks from which they derive therefore reflecting distinct geochemistry, NORMs included. This circumstance, therefore, suggests to pay great attention to the radioactivity of each single raw material since the final product might easily exceed radiation protection standards, independently of the addition of components like zircon sands, crucial to ceramic tile technology (see, for example, a similar discussion in our previous article devoted to alkali-activated materials, another type of composite material liable to exceedances in radiation protection standard metrics (Tositti et al. 2023)).

### Radiological parameters

The radiological hazard indices associated with the radionuclide composition of the building materials investigated are reported in Table 3 with the corresponding standard deviations. As discussed in paragraph 2.5, I_γ_ (the most important of these indexes for European law) should be less than the recommended value of 1; hence, the effective dose will not be higher than 1 mSv/year. Given the significant abundance of tiles and bricks in the database, 365 and 50 samples, respectively, and the final scope of these composites, the discussion on the radiological parameters will be mainly focused on them.

**Table 3:** Mean values and relative standard deviation of different hazard indices associated with the radioactivity of database samples

Material	Iγ	I_α_	Ra_eq_ [Bq/kg]	D_R_ [nGy/h]	AEDR [mSv/y]	H_in_	H_ex_
Brick	0.74 ± 0.16	0.34 ± 0.16	202 ± 45	181 ± 40	0.89 ± 0.20	0.74 ± 0.20	0.55 ± 0.12
Tiles	0.80 ± 0.20	0.49 ± 0.24	222 ± 57	25 ± 7	0.12 ± 0.03	0.88 ± 0.28	0.60 ± 0.15
Atomized	0.69 ± 0.14	0.34 ± 0.22	188 ± 42	168 ± 38	0.82 ± 0.19	0.70 ± 0.23	0.51 ± 0.11
Sand	1.13 ± 0.93	0.94 ± 1.17	320 ± 278	287 ± 247	1.40 ± 1.21	1.40 ± 1.41	0.86 ± 0.75
Zircon sand	13.22 ± 5.69	15.67 ± 7.43	3925 ± 1697	3495 ± 1529	17.13 ± 7.49	19.55 ± 8.82	10.61 ± 4.59
Clay	0.75 ± 0.12	0.32 ± 0.09	206 ± 32	180 ± 28	0.88 ± 0.14	0.74 ± 0.13	0.56 ± 0.09
Granite	1.10 ± 0.16	0.40 ± 0.11	291 ± 41	265 ± 40	1.30 ± 0.20	1.01 ± 0.17	0.79 ± 0.11

Different raw materials, such as clay and zircon sands, have different radiological impacts in the mixture. Regarding ceramic tiles, the use of zircon sand in the coating is indeed a well-known concern (Damonte et al. 2017). To avoid creating composites whose indices could exceed national limitations, attention is focused on activity concentration in raw materials. In recent years, the contribution from by-products has raised even more awareness of NORM presence in the construction industry and this has become significant when evaluating public exposure to radiation (Coletti et al. 2020).

Many radionuclides in natural decay chains are alpha emitters, and some (radon and progeny) are usually inhaled during indoor activities. The alpha index (I_α_) is then used to evaluate if the concentration of ^226^Ra in building material is below the recommended value of 200 Bq/kg (Krieger 1981). According to Table 3, the mean values of I_α_ with their standard deviations are 0.49 ± 0.24 for tiles, 0.34 ± 0.16 for bricks, 0.34 ± 0.22 for atomized materials, 0.94 ± 1.17 for sands, 0.32 ± 0.09 for clays, and 0.40 ± 0.11 for granite samples. This data shows that the internal hazard due to alpha particles is below the critical value for all these materials. On the contrary, zircon sands show much higher values, 15.67 ± 7.43, due to the significant ^238^U–^226^Ra concentration level in their composition.

As shown in Table 3, the mean value (± SD) of Ra_eq_ is 222 ± 57 Bq/kg for tiles, which is comparable with 183 ± 39 Bq/kg reported in (Barescut et al. 2009a) and 202 ± 45 Bq/kg for bricks. It is evident that Ra_eq_ values for each type of sample presented in this paper are lower than the recommended value of 370 Bq/kg (Beretka and Mathew 1985), except, clearly, for zircon sands.

The mean values of D_R_ are 25 ± 7 nGy/h and 181 ± 40 nGy/h for tiles and bricks, respectively, while the world average (population-weighted) indoor absorbed gamma dose rate reported in (European Commission: Directorate-General for Environment 2000) is 84 nGy/h. Consequently, the annual effective dose estimation AEDR reported in Table 3 is 0.12 ± 0.03 mSv/y for tiles and 0.89 ± 0.20 mSv/y for bricks. The worldwide average value of AEDR reported by UNSCEAR (UNSCEAR 2008) is 1 mSv/y. Therefore, the mean values of D_R_ and AEDR obtained for tiles are both below the global averages. On the other hand, the absorbed dose rate value for bricks is slightly above the global mean, while when it comes to the AEDR, the mean value is under the recommended threshold. These results show that the building materials considered are radiologically safe.

H_ex_ and H_in_ indexes as well are often used to characterize construction materials. The mean value calculated for H_ex_ is 0.60 ± 0.15 for tiles and 0.55 ± 0.12 for bricks, while for H_in_, the values are 0.88 ± 0.28 and 0.74 ± 0.20, respectively. Each of these mean values is below the limit, which is unity for both indexes.

**Fig. 4:**
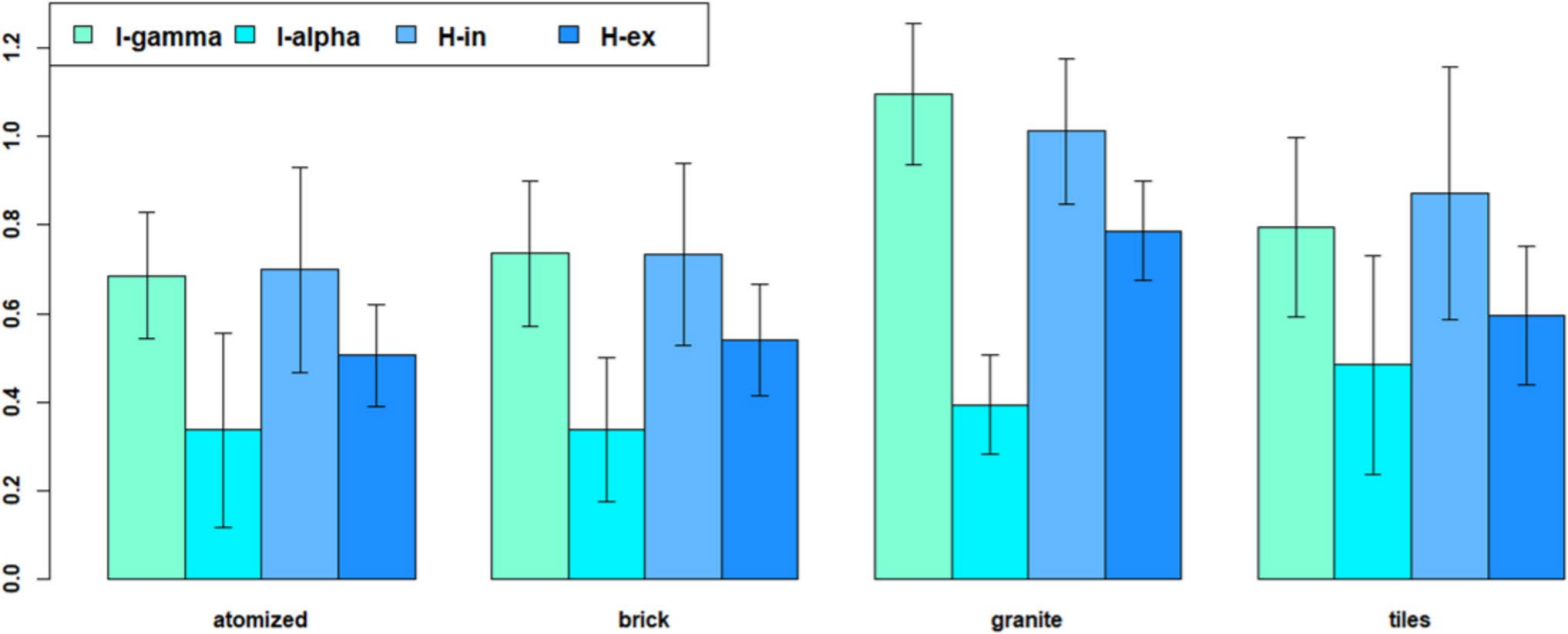
Mean values and the corresponding standard deviations of the dosimetric indices for building materials

A comparison between the different indexes is shown in Fig. 4 for the internal radiological indexes (I_α_ and H_in_); it can be noticed that H_in_ is more conservative than I_α_. Regarding the external quantities (I_γ_ and H_ex_), it shows that H_ex_ is less conservative than I_γ_.

**Fig. 5:**
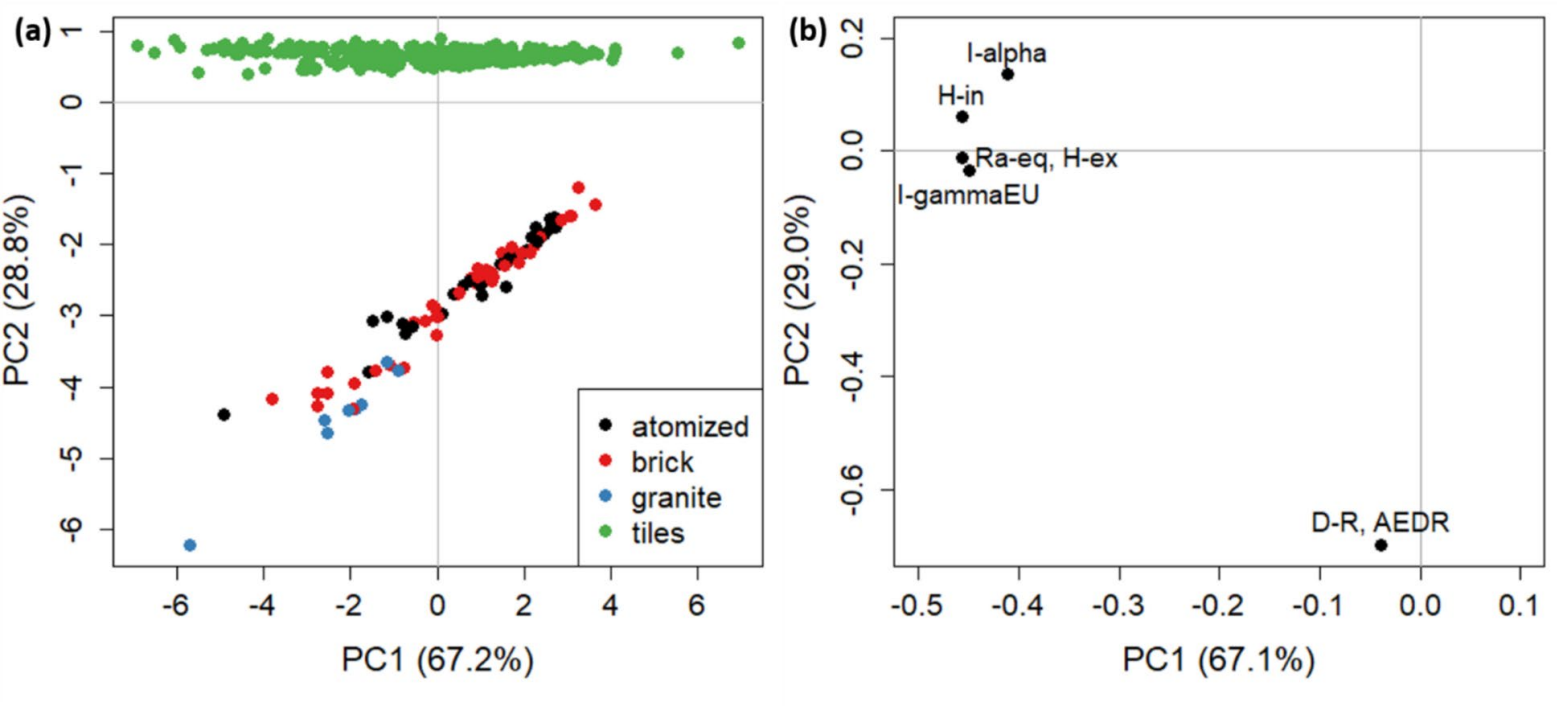
PCA of the indices for building materials: **a** scores plot, **b** loadings plot

A second PCA model was computed based on the indices previously introduced for the building materials (excluding clay, sands, and zircon sands). Figure 5 shows the scores (Fig. 5a) and the loadings (Fig. 5b) plot, where PC1 and PC2 represent 96.1% of total variance. It is interesting to note that the indices DR and AEDR, at high negative values of PC2, perfectly discriminate tiles from the other materials. As reported in Table 3 indeed, their mean values for tiles are much lower than that for other materials, reflecting the attention kept for their common inside use. Figure 5b shows also the closeness of I_γ_, Ra_eq_, and H_ex_, which are indices related to external exposition, while H_in_ and I_α_ to internal hazard.

## Conclusions

From the high-resolution HPGe gamma-ray spectrometry, analyses of 500 samples from common building materials and their raw components were carried out. Most of the samples reported originated in the Emilia-Romagna ceramic industry, and knowledge was formed that most materials investigated are radiologically safe in compliance with Euratom NORM thresholds, thanks to optimal balance among the components, usually characterized by significant natural variability, given how their NORM content proved consistently below the safety levels mandated by EU regulations.

The data reported and discussed in this paper contribute a substantial addition in terms of numbers and are in line with other datasets available in literature. They reflect the significant variability of NORM activity concentration levels in both geogenic and technically produced materials. This goes to stress further the need for accurate preliminary assessment of the radiometric levels of each component prior to utilization.

Concerning the values of radiometric indices obtained, their mean values proved to be systematically below the safety limits recommended confirming their safety for final use. The possible exceptions would be zircon sands; however, these are never used as such but always as a component, and the amounts used in the final products were correctly dosed thus preventing any hazard.

In conclusion, this work reflects the efforts applied in almost 15 years within the regionally important and strategic ceramic production field to comply with international radiation protection restrictions, and to increase awareness of current European dosimetric standards for building materials in general.

## Data Availability

The original contributions presented in the study are included in the article; further inquiries can be directed to the corresponding author.

## Footnotes

1 Atomized materials are intermediate products in the current porcelain grès production chain, produced by a high-temperature/high-pressure process where a slurry of various raw materials and pigments are wet-mixed by milling. The slurry (slip) is sent to the atomizer, where, due to further high-pressure/high-temperature processing, the droplets produced undergo swelling and explosion leading to fine fragmentation and drying; the final product is therefore very homogeneous and ready for the following steps.
